# A new method to reliably determine elastic strain of various crystal structures from atomic-resolution images

**DOI:** 10.1038/s41598-019-52634-3

**Published:** 2019-11-14

**Authors:** J. S. Chen, Y. Liu, Y. Zhai, T. X. Fan

**Affiliations:** State Key Lab of Metal Matrix Composites, School of Materials Science and Engineering, Shanghai Jiao Tong University, Shanghai, 200240 P.R. China

**Keywords:** Engineering, Materials science

## Abstract

Elastic strain engineering is an important strategy to design material properties in semiconductor and emerging advanced manufacturing industries. Recently, peak-pair method has drawn great attention compared to geometric phase analysis, owing to its precise determination of atom position at real space. Most current strain characterization methods estimate the local strain by comparing it with the related information from unstrained areas as reference. However, peak-pair method generated large errors in some cases because of the complexity of lower symmetric crystal structures, such as hexagonal structure. In this study, we introduce a new algorithm to overcome this limitation by directly comparing the atom positions with multiple references with different lattice symmetries. Furthermore, this new method is validated through several complicated crystal systems such as hexagonal, orthorhombic, monoclinic, and tetragonal structure, and returns expected values. This finding is essential to reliably determine the localized elastic strain with various crystal structures.

## Introduction

Localized elastic strain measurement is of great importance because local strain/stress are essential to tailor mechanical and physical properties of both semiconductor and composite materials. For instance, mechanical stress in electronic devices can lead to the formation of defects and change the band structure thus affects electronic performance^1^. In addition, in metallic materials, interfacial elastic strain field is one of the major factors that affect the formation and transition of interfacial dislocations^2,3^. Besides, the localized strain evolution in structural materials like multilayered composites can largely influence the mechanical properties^4,5^. Therefore, reliably determining the strain field at a high spatial resolution is crucial for the interface engineering and property optimization. Currently, various advanced two-dimensional strain mapping methods with a sub-micron resolution have been widely used, and can be classified into two main categories: pattern-based methods and image-based methods. One common feature of all these methods is that the measured strain is a relative value compared to the manually chosen unstrained reference region. As to the pattern-based method, it contains most of electron diffraction methods, including electron backscatter diffraction^6^, nano-beam electron diffraction^7,8^, nano-precession electron diffraction^9^ and convergent-beam electron diffraction^1,10^. These methods are insensitive to the lattice structure of the as-chosen materials because of the high symmetry of the diffraction features in the Fourier Space. The main limitation of these methods lies in that they are usually more time-consuming and has lower spatial resolution compared with that of the image-based methods.

Regarding the image-based methods, they include digital image correlation (DIC)^11,12^, geometric phase based method (GP)^13,14^ and peak-pair based methods (PP)^15,16^. Comparing with the real-time pattern-based methods, image-based methods are all post-processed so the images are usually preprocessed before determining the strain. They also estimate the local strain by comparing it with the related information from unstrained areas as reference. DIC is based on optical microscope (OM) and scanning electron microscopy (SEM) imaging techniques and the resolution can be up to sub-micro with advanced speckling techniques and algorithms^17,18^. Different from the other two methods, the strain measurement of this method only depends on the surface information, such as surface speckles. In comparison, both GP and PP methods are based on the high resolution (scanning) transmission electron microscopy (HR(S)TEM) images with the atomic resolution. In addition, GP estimates the strains from Fast Fourier transform (FFT) of HR(S)TEM images, whereas PP directly estimates the strain in real space. This difference grants GP a pivotal advantage over PP of its universality in various crystal structures^19–22^, which makes it more widely accepted. Nevertheless, PP has its own advantages in several aspects. First, it employs more image information rather than GP and the noise reduction process can be properly controlled. Secondly, PP has greater capability to operate in image pre-processing and detailed strain analysis in confined areas. Thirdly, the strain value near the dislocation core or other local disturbance may cause some confusion in GP. For example, in Zhu’s work^23^, GP method was applied to measure the strain map of a simulated heterogeneous interface between SrTiO_3_ and MgO. Periodic strain dipoles are observed in the associated strain maps. However, since the two different lattices are simply put together without introducing any misfit dislocations along the interface, these strain convergent regions are caused by the phase jumps between the two different lattice parameters. Similar interfacial lattice strain in unstrained regions are observed in In_0.7_Ga_0.3_As-AlAsSb system due to the artefacts in GP^24^. Consequently, when such strain concentrations are observed in real image, it will be hard to assure whether it comes from real defects or just from the inherent structure-induced errors. As a result, this limitation of GP makes PP more promising in defects characterization.

**Figure 1:**
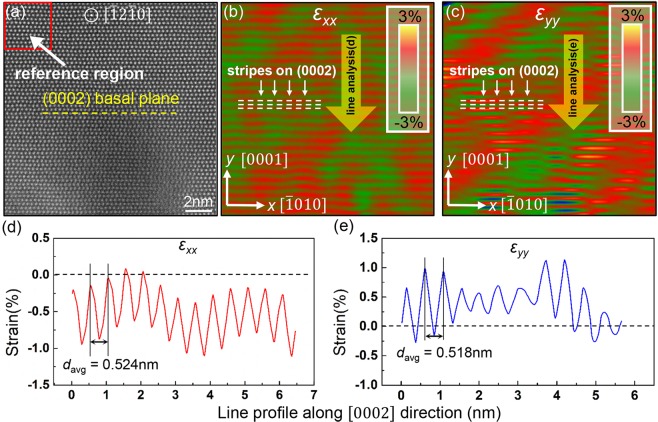
The original PP method applied on Mg structure. (**a**) An HRSTEM image of unstrained Mg viewing from [$\bar{1}$2$\bar{1}$0] direction. The {0002} basal plane is denoted with yellow dashed line. (**b**,**c**) The measured strain maps of *ε*_*xx*_ and *ε*_*yy*_ using original PP. Apparent strain stripes can be observed along the {0002} basal plane (as denoted in white dashed lines). Line analysis results along the yellow arrows are shown in (**d**,**e**). The average interval space *d*_*avg*_ between the two adjacent strain peaks are 0.524 nm and 0.518 nm for *ε*_*xx*_ and *ε*_*yy*_, respectively. Both values are close to the *c* length of Mg (0.521 nm).

Nowadays, PP works well on all the highly symmetrical crystal systems, such as Ge/Si^25^, InAs/GaAs^16^, InAs/GaSb^26,27^ and AlSb/GaAs^28^ to study the strain field at the heterogeneous interface. One common characteristic of these material systems are either face-centered-cubic (FCC) or body-centered-cubic (BCC) structures. However, few studies have applied this method in more complex structures, for example, in non-cubic crystal structures. The interface-associated strain field is of great research value since heterogeneous interface is one of the critical factors to mediate the mechanical properties. Thus, it’s necessary to firstly validate PP’s feasibility in a representative crystal structure with lower symmetry, such as hexagonal-closed-packed (HCP) Mg. Figure 1a is an HRSTEM image of unstrained Mg observed along $[\bar{1}2\bar{1}0]$ zone. Figure 1b,c are the measured strain maps using original PP. The unstrained reference region is set on the top-left of the image. The *x* axis and *y* axis are set along $[\bar{1}010]$ and [0001] direction, respectively. Apparent strain stripes are observed parallel to {0002} basal planes. Line analysis along the yellow arrows are shown in Fig. 1d,e. The strain values manifest strong periodic feature along the [0001] axis with a peak value of ~−1% for *ε*_*xx*_ and ~1% for *ε*_*yy*_. The period length *d*_*avg*_ for *ε*_*xx*_ and *ε*_*yy*_ are 0.524 nm and 0.518 nm, close to the *c* length of Mg. These results confirm that the strain stripes appear alternatively on the {0002} basal planes. It also indicates that the conventional idea of choosing only one reference region for strain mapping is not sufficient for conventional PP method for lower symmetrical lattice.

Therefore, in this work, we re-evaluated this problem and provided a possible solution to expand its applicability in most of the crystal systems. We modified the original peak-pair method with a new structural-reference related algorithm and developed a more flexible modified peak-pair method (m-PP). This m-PP directly compares the atom positions with multiple references with different lattice symmetries. In addition, we validated this new method through several complicated crystal structures such as hexagonal, orthorhombic, monoclinic, and tetragonal structure, and returns expected values. In general, our work provides a new method for reliable determination of elastic strain from various crystal structures at atomic scale resolution, which shows great potential in elastic strain engineering related field in both semiconductor and composites industry.

## Results and Discussion

### Applying m-PP on HCP Mg

**Figure 2:**
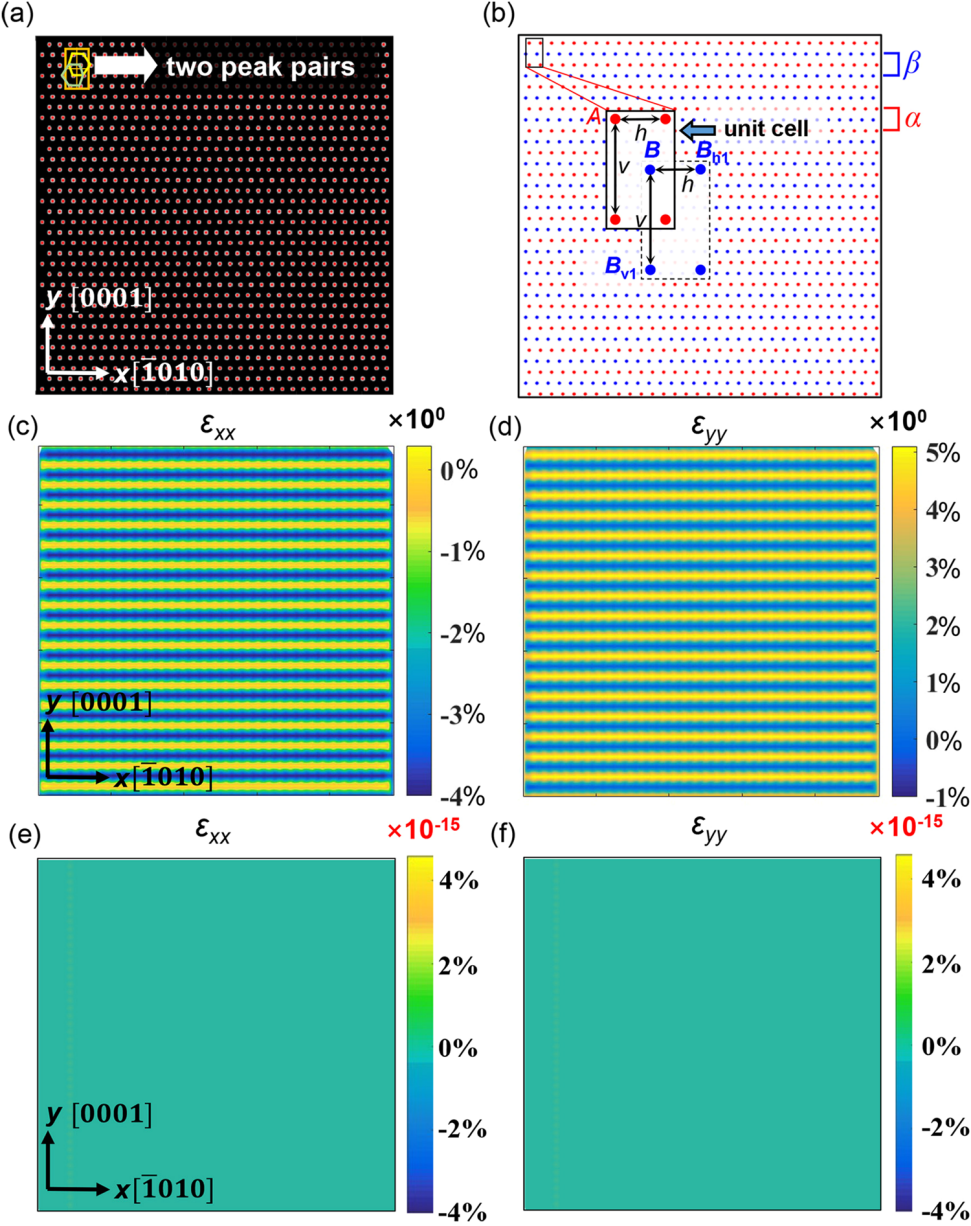
Different strain calculation results of the same non-strained Mg lattice using single reference (original PP) and two references (m-PP). (**a**) The simulated HCP lattice and the peak detection result. (**b**) The atom layer separation process. The unit cell is denoted on the top left and enlarged to show the separating process. The red dots are *α* layer atoms and blue dots are *β* layer atoms. The horizontal distance *h* and vertical distance *v* are used to search for the atoms belong to the same layer. (**c**,**d**)*ε*_*xx*_, *ε*_*yy*_ strain maps calculated using single reference, respectively. (**e**,**f**) *ε*_*xx*_, *ε*_*yy*_ strain maps calculated using two references. It shows apparently that by combining the two references, the minimum strain value of a non-strained Mg lattice can be achieved, which is 15 orders of magnitude smaller than using only one reference.

For better illustrating the difference between the original PP and m-PP, we built an unstrained perfect lattice projection map to simulate the HCP Mg HRSTEM image on $[\bar{1}2\bar{1}0]$ zone, as shown in Fig. 2a. The atoms are shown as bright dots with a brightness distribution inversely proportional to the distance from the center. The red dots are the detected peaks for each atom using a local maximum algorithm. It should be mentioned that the peak detection process will be much more complex for real image, including real space and Fourier Space noise reduction and distortion correction. However, these factors are not taken into consideration here because the choice of reference in nature is only related to structural factors. Figure 2b is the atom layer separation results. The different layers (*α *and *β*) are colored as blue and red, respectively. Horizontal (*h*) and vertical (*v*) lattice parameters are defined in the unit cell (top left) to classify the atoms into different layers. To be specific, the *α* layer atoms will be located starting from point A in searching steps of *h* (horizontal) and *v * (vertical) within a certain deviation range. The same process is repeated on point B to separate *β* layer atoms. For example, *B*_h1_ and *B*_v1_ are the two nearest B layer atoms.

The next step is strain calculation using the new algorithm. The original PP uses only one set of reference parameters to calculate the strain tensor. The results are shown in Fig. 2c,d. The *x* axis and *y* axis are along $[\bar{1}010]$ and [0001] direction, respectively. Similar strain stripes are observed for both *ε*_*xx*_ and *ε*_*yy*_. The strain layer with a smaller absolute value in both directions is zero, corresponding to the same layer where the reference lattice is located. The strain value in the neighboring layer is −4% for *ε*_*xx*_ and 5% for *ε*_*yy*_, respectively. Since the built lattice is unstrained, this large strain value is definitely erroneous due to the mismatch of the reference and the calculated atom layer. The signs of the strain value may be opposite when the reference peak pair is located in the other atom layer. It should also be noticed that the peak strain value in this simulated image is different from the real image in Fig. 1. This may be attributed to several aspects: (1) the atom positions in real image is slightly different from the simulated image due to the experimental errors such as scanning effect. (2) The difficulty of peak detection process in real image is much higher than simulated image due to the ununiformed atom brightness distribution. (3) The reference parameters in real image is an average of the two different sets of reference peak pairs. While in this simulated image, parameters from only one peak pair is used. In addition, the strain value has been smoothed to different extends when mapping. However, since all these strain values are relative values compared to the reference, only the distribution features are considered here and the stripes are enough to illustrate the problem of original one-set reference. The modified method uses two sets of reference peak pairs to calculate the strain tensor separately, as shown in Fig. 2a. The corresponding *ε*_*xx*_ and *ε*_*yy*_ strain maps are shown in Fig. 2e,f. It is obviously that the strain is much more uniform and the magnitude is in the range of 10^−15^, which may be ignored due to precision fluctuation. These results indicate that the modified PP is applicable on HCP lattice.

### Applying m-PP on complex crystal structures

**Figure 3:**
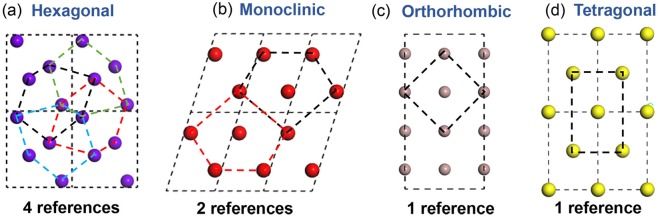
Schematic of [010] atomic projection of different structured Ga. (**a**) Hexagonal. (**b**) **Monoclinic**. (**c**) Hexagonal. (**d**) Tetragonal. All the atomic projections are along [010] direction. The dashed lines show the choice of reference peak pairs.

**Figure 4:**
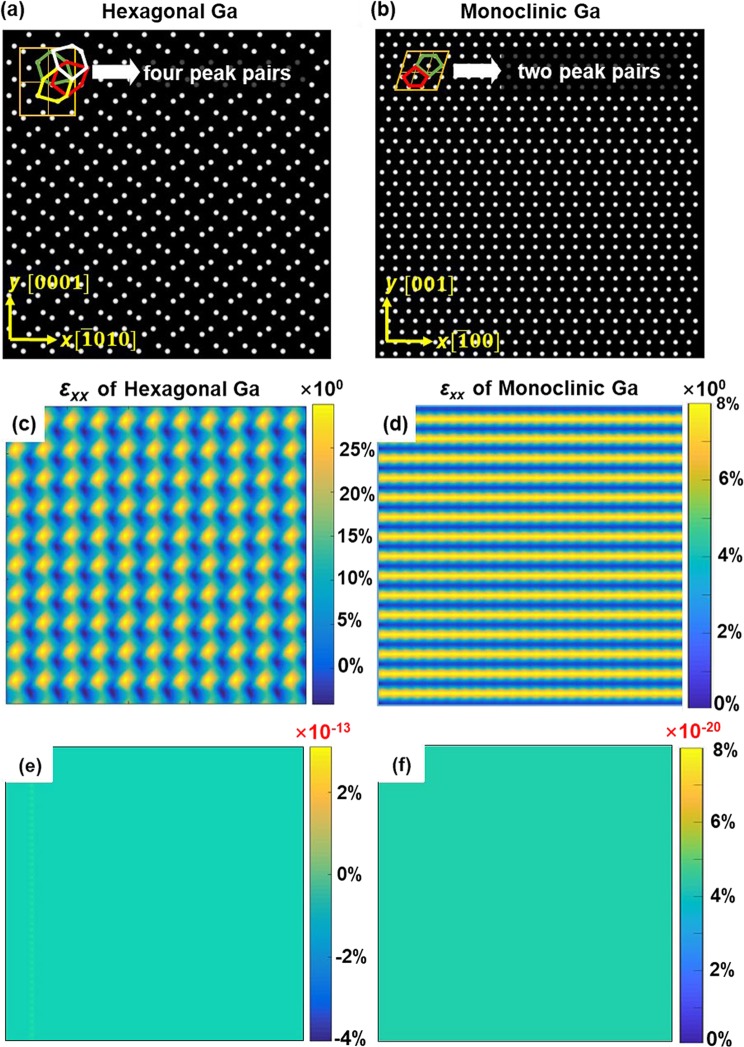
Results of m-PP applied on non-strained Ga. (**a**,**b**) The simulated hexagonal and monoclinic lattices of Ga. The peak pairs are denoted in different colors. (**c**,**d**) *ε*_*xx*_ strain maps calculated using only one reference. (**e**,**f**) *ε*_*xx*_ strain maps calculated using all the necessary references (four for hexagonal Ga and two for monoclinic Ga). It should be noticed that the strain magnitude of (**e**,**f**) are 10^−13^ and 10^−20^, respectively. The results show that the new method is applicable on complex crystal structures once all the references are chosen properly.

In order to validate m-PP through several complicated crystal structures, here we choose four different structured Ga lattices for close comparison. As shown in Fig. 3, these projections are all along the [010] zone for the different structures. Figure 3a is another hexagonal structured Ga lattice and there are four atom layers in each unit cell. Each atomic layer within single unit cell does not have a simple geometric symmetry about the *x* axis or *y* axis, thus four different reference peak pairs (marked in different colored hexagons) are needed in this case. Similarly, Fig. 3b is a monoclinic structured Ga lattice with two non-equivalent atom layers. Figure 3c, d are the orthorhombic and tetragonal Ga lattices, respectively. These two lattices can be classified into the same category. The orthorhombic Ga has four atom layers in each unit cell. However, the adjacent two layers of atoms are arranged exactly the same, and the atoms are equally spaced between the layers. As a result, the unit cell can be simplified into a one-layer structure and only one reference lattice is necessary. Similarly, the tetragonal lattice has three different atom layersbut only one reference is needed. From the above discussion, it is clear that the number of reference peak pairs not only depends on the number of atom layers but also the lattice symmetry. In addition, if the atomic layers are evenly distributed in one direction, the number of required reference lattices can also be greatly reduced. Figure 4a and Fig. 4b are the simulated HRSTEM image of these two Ga crystal structures (hexagonal and monoclinic). The corresponding *ε*_*xx*_ strain maps using the original PP and m-PP are shown in Fig. 4c,d and Fig. 4e, f, respectively. Similar to HCP Mg, regular periodic strain patterns can be observed in Fig. 4c,d and the maximum values are about 25% for hexagonal Ga and 8% for orthorhombic Ga. In these cases, only one reference is used and there is a large mismatch between the reference peak pair and the atoms. It should be noticed that although there are four different atom layers in hexagonal Ga, four separate strain layers can't be observed clearly. This is not surprising as the different peak pairs can be symmetrical (as shown in Fig. 3a) so that strain errors may cancel with each other in some directions. In comparison, after applying different peak pairs, ideal strain value can be obtained with a range within ± 5% × 10^−10^ for both cases. This indicates that this modified method is applicable for even more complicated structures once the unit cell is defined and the peak pairs are chosen properly. The key point of this new method is defining the unit cell of the HRTEM/HRSTEM image manually and choosing the characteristic points so that the different atom layers can be distinguished. It should be mentioned that the algorithm for strain calculation is not unique and any other effective algorithm can be used to combined with m-PP. The main limitation of the method is that it will loss efficiency when the lattice is too complex to differentiate  the atoms layers. In these cases, we can use cross-correlation to simplify the lattice structure before applying this method.

**Figure 5:**
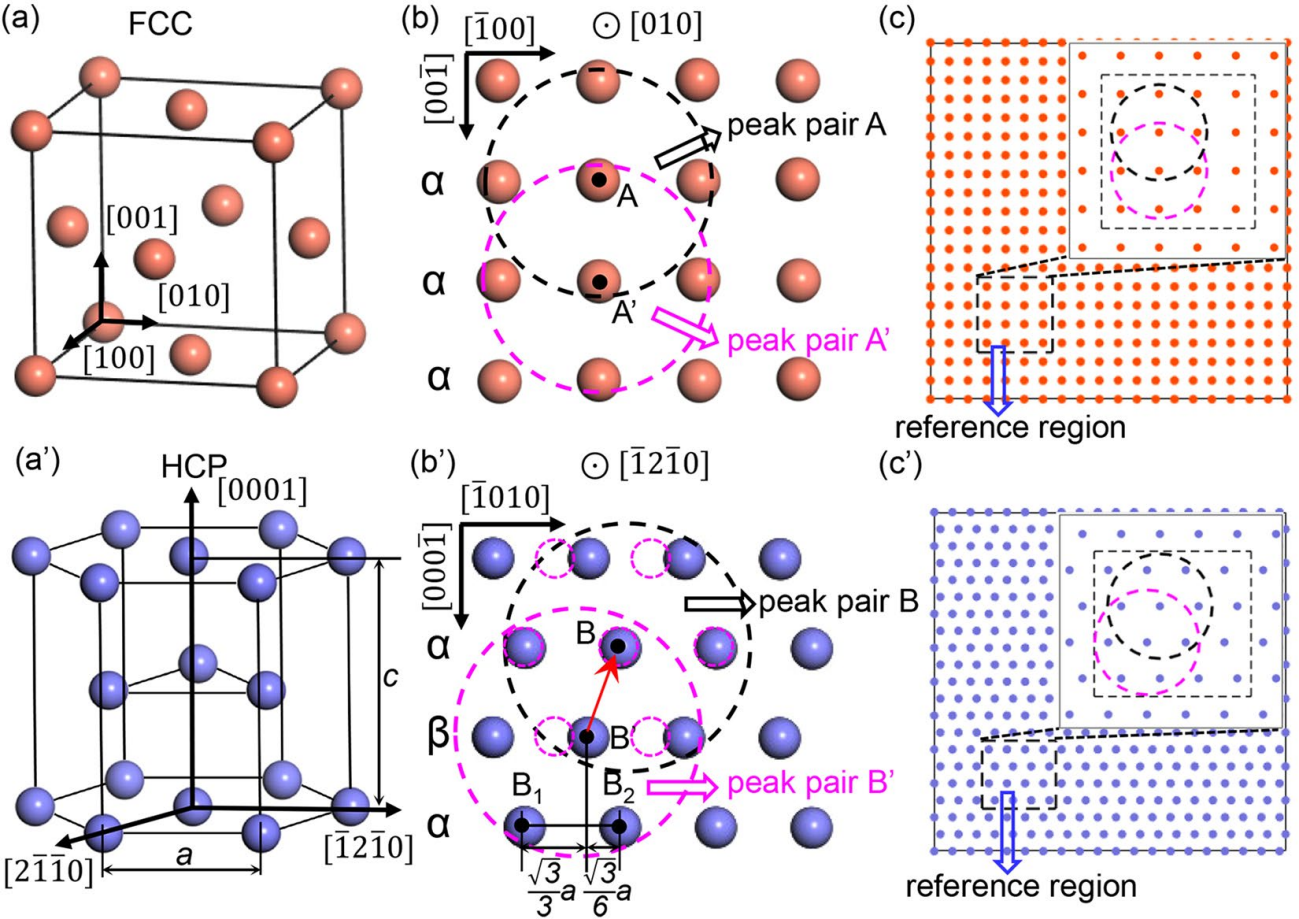
Comparison of the atom arrangements and peak pair selection of FCC and HCP structures. (**a**,**a’**) are the crystal cells of FCC and HCP structures, respectively. (**b**) The atomic projection of FCC structure along [010] direction. (**b’**) The atomic projection of HCP structure along $[\bar{1}2\bar{1}0]$ direction. The black and purple dashed circles show two reference peak pairs. Because of the high symmetry of FCC, the two peak pairs A and A’ in (**b**) are actually the same. However, as a result of the lower symmetry in HCP structure, the lateral distance between B’ and B_1_ is twice of that between B’ and B_2_. Hence the two reference peak pairs in (**b’**) are of mirror symmetry. By translating the peak pair B’ to B along the red arrow, it shows that the two sets of atoms can’t completely coincide. (**c**,**c’**) are schematics of the atomic projection and reference peak pairs in (**b**,**b’**) achieved by MATLAB. The dashed black rectangle gives the selected reference area.

## Conclusion

In sum, we have modified the image-based peak-pair method by defining various necessary reference-matrix for nonsymmetric crystal structures, and then calculated the strain tensor of each atom separately. The new method has been demonstrated in HCP Mg and also in other complex structures such as orthorhombic, monoclinic and tetragonal structures. This method is compatible with most metal, ceramic and semiconductor materials, and possesses potential applications in strain-engineering researches.

## Methods

### Materials and algorithm

Figure 5 gives the schematic of the undergoing mechanism of the strain stripes caused by the original one-set peak pair choice in PP. We compared FCC and HCP structures to show the crystal structure’s effect on the result. Projection zone is set parallel to [010] direction for both FCC and HCP. Figure 5a is a typical FCC unit cell. The atomic projection map is shown in Fig. 5b. Similarly, Fig. 5a’,b’ are the unit cell of HCP and its projection along $[\bar{1}2\bar{1}0]$ direction. Like most of the other strain measuring methods, the first step of PP is choosing an unstrained region as a reference to get the basic parameters of the lattice for further measurement. Here we consider an extreme case that the reference region size equals to the size of exact one peak pair. In real situations, the average value of the parameters in the reference region will be used. However, this extreme case is more persuasive to illustrate the problem. The purple and black dashed circles centered on A and A’ in Fig. 5b are the two possible reference peak pairs for FCC. The parameters obtained from any of these two peak pairs will be treated as reference parameters to define the strain tensor of every other atom (the details about the strain algorithm will be discussed later). Fortunately, due to the high symmetrical arrangement of atoms in FCC, these two peak pairs are actually the same. As a result, peak pairs from any atom layer in the unstrained region is an efficient reference. Figure 5c shows the schematic of the atom arrangement of a perfect FCC structure on [010] zone. The reference peak pair parameters are independent on its location and which atom layer it belongs to. In another word, parameters from any single peak pair or the average value of all the peak pairs in the reference region in Fig. 5c are exactly the same. Thus, the original PP can be applied directly on FCC structures. However, the case becomes more complicated when it comes to HCP structures. As shown in Fig. 5b’, the atomic projection along $[\bar{1}2\bar{1}0]$ shows an alternative feature of ***α***, ***β, α***……. This indicates that the atoms in ***α*** layer and ***β*** layer are embedded in different environment. To be specific, the horizontal distance between one atom and the nearest two atoms in the neighboring layer are $\frac{\sqrt{3}}{6}a$ and $\frac{\sqrt{3}}{3}a$, respectively (Fig. 5b’), where *a* is the lattice parameter. Two randomly chosen peak pairs centered on B and B’ are denoted in black and purple dashed circles, respectively. By overlapping B and B’, these two references cannot coincident with each other precisely with a mirror symmetry. Thus, the conventional method of randomly choosing one reference region and simply using the average lattice parameters will produce an incorrect result in this case, as discussed in Fig. 1. To deal with this, in this study, we improve the original method by defining one set of reference lattice for each atom layer (Fig. 5c’). The program separates the different layers automatically after the unit cell is defined and uses the corresponding reference for strain calculation.

**Figure 6:**
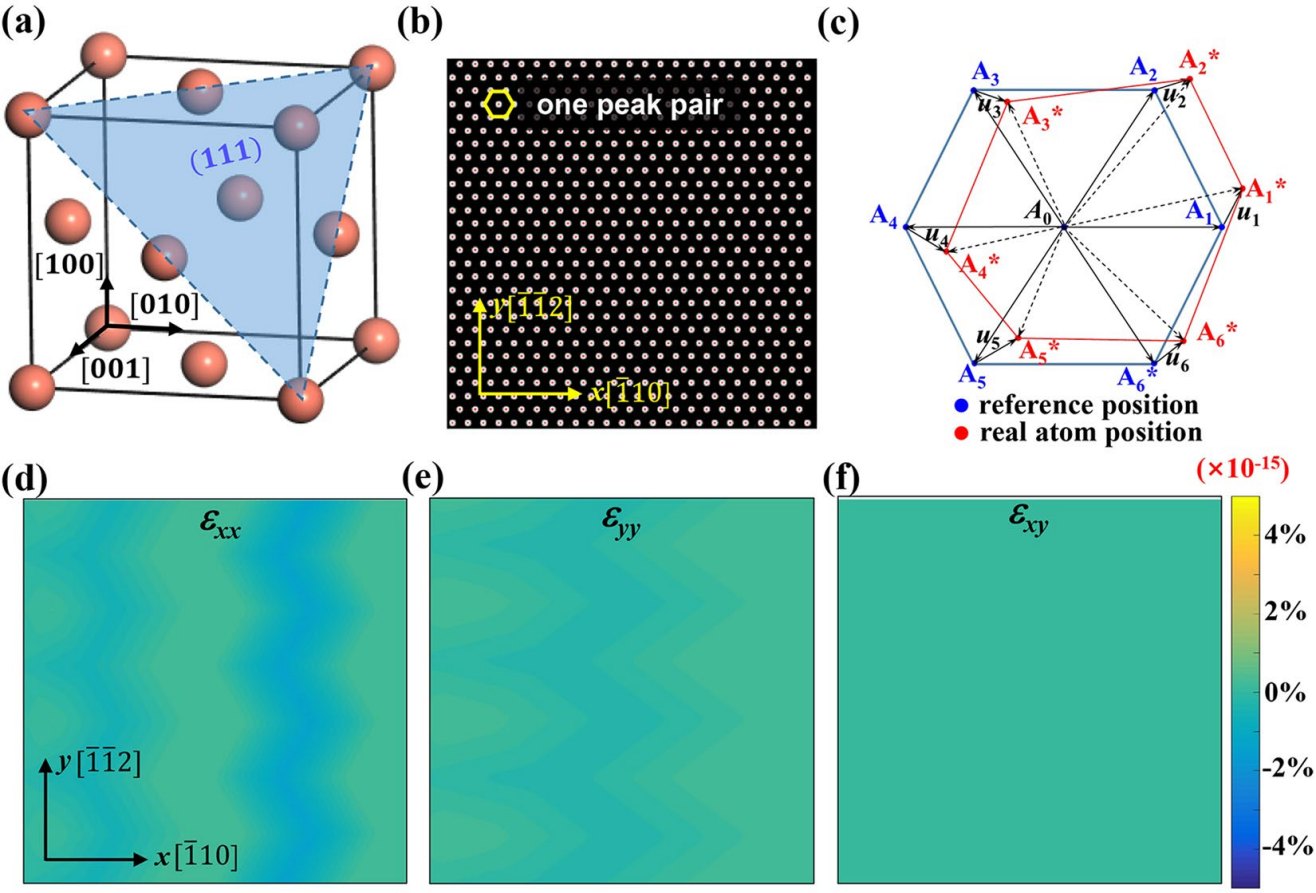
The schematic illustration of the new strain calculation algorithm applied in the [111] atomic projection of FCC structure. (**a**) The lattice structure of FCC. (**b**) The simulated HRSTEM images of FCC structure on [111] zone. (**c**) The displacement vectors of the six nearest atoms around the central atom. (**d**,**e**,**f**) The *ε*_*xx*_, *ε*_*yy*_ and *ε*_*xy*_ strain maps of unstrained FCC lattice.

The next step is using the reference parameters to calculate the strain tensors of each atom. Here we used a new algorithm different from origin PP in two aspects. It was firstly released by Hoagland *et al*.^29^ in a three-dimensional form and was deduced to a two-dimensional from by N. Li *et al*. to use in FCC TiN^30^. The first difference is the peak pair defining process. In original PP algorithm, only two neighboring atoms are chosen for each peak pair. In this work, atoms within a specific radius from the center atom are chosen as peak pair atoms, as shown in Fig. 5b,b’. Using more atoms in one peak pair is an efficient way to reduce the effect of noise and get a smoother strain value. The second difference is the strain tensor determination algorithm. The algorithm we applied in this work uses the offset vectors of the six neighboring atoms to calculate the strain tensor of each central atom using the least squares determination of the strain ellipsoid. Figure 6 is a schematic of its application in FCC structure. Figure 6a is the structure of FCC lattice, the marked (111) plane is perpendicular to the projection direction. The red dots in Fig. 6b are the projection of the strained lattice on the [111] zone. The set of seven blue dots and seven red dots in the dashed black circle is the selected reference peak pair cell and real peak pair cell, respectively. Figure 6c shows the magnified details inside the black circle. *A*_1_~*A*_6_ are the reference atoms and *A*_1_^*^ ~ *A*_6_^*^ are the corresponding strained lattices. By overlapping these two peak pairs at *A*_0_, the offset vectors of each surrounding atom (*A*_i, i=1 ~6_) can be determined as *u*_i, i=1~6_. Then the strain tensor:

$$\varepsilon =(\begin{array}{c}{\varepsilon }_{xx}\\ {\varepsilon }_{yy}\\ {\varepsilon }_{xy}\end{array}),$$

at atom *A*_0_ can be calculated through Eq. (1):

1 $$\varepsilon ={N}^{-1}\cdot Q$$

where *N* is the structural factor matrix of the lattice, which is only associated with the lattice structure and the shape of reference peak pair. In this FCC structure with a [111] projection, it is calculated to be $$(\begin{array}{ccc}2.25 & 0.75 & 0\\ 0.75 & 2.25 & 0\\ 0 & 0 & 0.75\end{array})$$. *Q* is the displacement matrix related to both the structure parameters and the offset vectors *u*_*i*_. The details of the algorithm are shown in Supplementary Materials. Figure 6d–f are the calculated *ε*_*xx*_, *ε*_*yy*_, *ε*_*xy*_ strain maps of an unstrained FCC lattice. The *x* axis and *y* axis are set along $[\bar{1}10]$ and $[\bar{1}\bar{1}2]$ direction. All of these strain maps show a uniform distribution of strain at the magnitude of 10^−15^, which can be treated as strain free considering the precision fluctuation. This indicates that the new peak defining method with the associated strain algorithm works well in FCC structure. As discussed above, the main difference between HCP and FCC is the number of needed references for strain calculation. Firstly, the *N* matrix of the two references should be determined separately. Fortunately, both *N* matrixes are calculated to be:

$$(\begin{array}{ccc}2/{(3{r}^{2}+1)}^{2} & (4+8/3{r}^{2}+4+6{r}^{2}/3{r}^{2}+1) & 0\\ (4+8/3{r}^{2}+4+6{r}^{2}/3{r}^{2}+1) & 36{r}^{4}/{(3{r}^{2}+1)}^{2} & 0\\ 0 & 0 & (4+8/3{r}^{2}+4+6{r}^{2}/3{r}^{2}+1)\end{array}),$$

where *r* is the *c/a* ratio of the HCP material. Thus, only one *N* is needed here. The same value of *N* matrix for the two kinds of peak pairs is due to their mirror symmetry about the *y* axis. As a result, in this case, the necessity of two reference lattices for HCP is mainly reflected in the determination of the atomic offset vector *u*_i_, which determines the displacement matrix *Q*. However, this isn’t always true when the lattice is more complicated. In another word, in some other crystal structures like hexagonal Ga, the *N* matrix of each reference should be defined independently. This function has been implemented automatically in our improved algorithm, as long as different reference peak pairs are defined. The comparison of results between the original and modified methods are demonstrated in the results and discussion section.

## Supplementary information

Supplementary Materials.

## Data Availability

All data generated or analyzed during this study are included in this published article. The strain calculation codes of the current study are available from the corresponding author per request.
