# Supplementary material for: A new method to reliably determine elastic strain of various crystal structures from atomic-resolution images
Source: Sci Rep. 2019 Nov 14;9:16399. doi: 10.1038/s41598-019-52634-3 (PMC6856106; doi:10.1038/s41598-019-52634-3)
Supplement: Supplementary file 1 — Supplementary Materials. [file 41598_2019_52634_MOESM1_ESM.docx]

**A new method for** **reliable determination of elastic strain from** **nonsymmetric crystal structure from atomic resolution images**

J.S. Chen^1^, Y. Liu^1^*, Y. Zhai^1^, T.X. Fan^1^*

^1^ State Key Lab of Metal Matrix Composites, School of Materials Science and Engineering, Shanghai Jiao Tong University, Shanghai 200240, P.R. China.

* Corresponding authors: [yliu23@sjtu.edu.cn](mailto:yliu23@sjtu.edu.cn); [txfan@sjtu.edu.cn](mailto:txfan@sjtu.edu.cn).

**Details of the strain calculation algorithm**

The strain vector $\overrightarrow{u}=\left[\begin{matrix} u_{1} \\ u_{2} \end{matrix}\right]$ of a single point in the 2D coordinate system (X, Y) can be described as $\overrightarrow{u}=\overrightarrow{D}\overrightarrow{X}$, where $\overrightarrow{D}$ is the derivative matrix:

$\overrightarrow{D}=\left(\begin{matrix} \frac{\partial u_{1}}{\partial X} & \frac{\partial u_{1}}{\partial Y} \\ \frac{\partial u_{2}}{\partial X} & \frac{\partial u_{2}}{\partial Y} \end{matrix}\right)$ (1)

$\overrightarrow{X}=\left[\begin{matrix} X \\ Y \end{matrix}\right]$ (2)

and the strain tensor component:

(3)

After we detecting the position of each atom in the HR(S)TEM, the peak pair then can be defined manually. For example, the central atom and its nearest 6 neighbors can be treated as a peak pair (as shown in **Figure 5b**). As a result, the next step is to determine the strain tensor of the central atom in each peak pair.

Suppose that there are $N$ neighbors around each central point in the unstrained region, let $\alpha^{R}$ be the standard distance between the $\alpha^{th}$ neighbor and the central atom. Then we have:

$\alpha^{R}\text{=}\sqrt{\alpha^{X_{1}^{2}}+\alpha^{X_{2}^{2}}}$ (4)

Similarly, we can obtain the distance in the strained region as $\alpha^{r}$, so the relative strain corresponding to the $\alpha^{th}$ neighbor can be derived as:

$\alpha^{\varepsilon}=\frac{\alpha^{r}}{\alpha^{R}}-1$ (5)

Let be the displacement of the $\alpha^{th}$ atom from the original position, we have:

$\alpha^{R}(1+\alpha^{\varepsilon})=\sqrt{(\alpha^{X_{1}}\text{+}\alpha^{u_{1}})^{2}+(\alpha^{X_{2}}\text{+}\alpha^{u_{2}})^{2}}$ (6)

Neglecting the squared terms, we have

(7)

Combine (3), (5) and (7), and let $\gamma_{12}\text{=}2\varepsilon_{12}$, we have:

(8)

Let $\alpha^{l}=\frac{\alpha^{X_{1}}}{\alpha^{R}}$, $\alpha^{m}=\frac{\alpha^{X_{2}}}{\alpha^{R}}$, (8) becomes:

$\alpha^{\varepsilon}\text{=}\alpha^{l^{2}}\varepsilon_{11}+\alpha^{m^{2}}\varepsilon_{22}+\alpha^{l}\alpha^{m}\gamma_{12}$ (9)

Let’s determine the least mean square (LSD) values of the strain components at central point. To do that, subtract the right hand side of (9) from the left hand side, square it, and sum over all of the nearest neighbors:

$L=(\alpha^{\varepsilon}\text{-}\alpha^{l^{2}}\varepsilon_{11}\text{-}\alpha^{m^{2}}\varepsilon_{22}\text{-}\alpha^{l}\alpha^{m}\gamma_{12})^{2}$ (10)

Let $\frac{\partial L}{\partial\varepsilon_{ij}}=0$, we can get the strain tensor by solving the equation:

$$N\varepsilon=Q$$

Where$N=\left(\begin{matrix} \sum\alpha^{l^{4}} & \sum\alpha^{l^{2}}\alpha^{m^{2}} & \sum\alpha^{l^{3}}\alpha^{m} \\ & \sum\alpha^{m^{4}} & \sum\alpha^{l}\alpha^{m^{3}} \\ & & \sum\alpha^{l^{2}}\alpha^{m^{2}} \end{matrix}\right)$, , $Q=\left[\begin{matrix} \sum\alpha^{\varepsilon}\alpha^{l^{2}} \\ \sum\alpha^{\varepsilon}\alpha^{m^{2}} \\ \sum\alpha^{\varepsilon}\alpha^{l}\alpha^{m} \end{matrix}\right]$.
